# Low Temperature Effect on the Endocrine and Circadian Systems of Adult *Danio rerio*

**DOI:** 10.3389/fphys.2021.707067

**Published:** 2021-11-24

**Authors:** Cristhian D. Sua-Cespedes, Daniela Dantas David, José A. Souto-Neto, Otoniel Gonçalves Lima, Maria Nathália Moraes, Leonardo V. Monteiro de Assis, Ana Maria de Lauro Castrucci

**Affiliations:** ^1^Laboratory of Comparative Physiology of Pigmentation, Department of Physiology, Institute of Biosciences, University of São Paulo, São Paulo, Brazil; ^2^Laboratory of Neurobiology, Department of Physiology and Biophysics, Institute of Biomedical Sciences, University of São Paulo, São Paulo, Brazil; ^3^Center of Brain, Behavior and Metabolism, Institute of Neurobiology, Lübeck University, Lübeck, Germany; ^4^Department of Biology, University of Virginia, Charlottesville, VA, United States

**Keywords:** *Danio rerio*, temperature, hormones, clock genes, hormone receptors

## Abstract

The control of the biological rhythms begins with the activation of photo- and thermosensitive cells located in various organs of the fish such as brain, eye, and skin, but a central clock is still to be identified in teleosts. Thermal changes are stressors which increase cortisol and affect the rhythm of other hormones such as melatonin and growth hormone (GH), in both endo- and ectothermic organisms. Our aim was to investigate how temperature (23°C for 6 days) lower than the optimal (28°C) modulates expression of several gene pathways including growth hormone (*gh1*) and its receptors (*ghra, ghrb*), insulin-like growth factor1 (*igf1a, igf1b*) and its receptors (*igf1ra, igf1rb*), cortisol and its receptor (*gr*), the limiting enzyme of melatonin synthesis (arylalkylamine N-acetyltransferase, *aanat*) and melatonin receptors (*mtnr1aa, mtnr1bb*), as well as their relationship with clock genes in *Danio rerio* in early light and early dark phases of the day. Lower temperature reduced the expression of the hormone gene *gh1*, and of the related receptors *ghra, ghrb, igf1ra*, and *igf1rb*. Cortisol levels were higher at the lower temperature, with a decrease of its receptor (*gr*) transcripts in the liver. Interestingly, we found higher levels of *aanat* transcripts in the brain at 23°C. Overall, lower temperature downregulated the transcription of hormone related genes and clock genes. The results suggest a strong correlation of temperature challenge with the clock molecular mechanism and the endocrine systems analyzed, especially the growth hormone and melatonin axes, in *D. rerio* tissues.

## Introduction

A variety of physiological and biochemical processes displays a daily rhythmic profile which is adjusted by environmental factors such as temperature and light, major temporal cues and first order *zeitgebers* (time-giver in German) (Aschoff, 1965; West and Bechtold, 2015). In the natural environment, temperature has a close relationship with the light-dark cycles, as the thermo-phase (high temperature) usually coincides with the photo-phase, and the cryo-phase (low temperature) coincides with the scoto-phase (López-Olmeda and Sánchez-Vázquez, 2011).

Early studies in *Danio rerio* showed that oscillations in the clock components were very similar to the mouse, and occurred in all tissues examined, both *in vivo* and *in vitro* (Lahiri et al., 2005). However, it’s important to notice that the peak of clock genes occur in antiphase in these species. In addition to the canonical light effects, temperature variation also promotes changes in the clock molecular core of *D. rerio* tissues and cells (Lahiri et al., 2005; Jerônimo et al., 2017).

In *D. rerio*, the protein products of *clock* and *bmal1* genes (peak at dusk) activate another group of clock genes and clock-controlled genes, through the binding of Clock:Bmal heterodimers to E-box elements in the target genes, including *period (per)* and *cryptochrome (cry)*. The genes *per* and *cry* are transcribed (peak at dawn), translated and transported into the nucleus as Per:Cry heterodimers which inhibit the transcription of Clock:Bmal complex. This complex is reactivated after degradation of Per:Cry. This cycle is adjusted to a 24-h period by the light-dark cycle and represents the molecular mechanism by which the organism keeps track of time (Lahiri et al., 2005; Vatine et al., 2011). The rhythm amplitude of some clock gene expression in *D. rerio* is strongly influenced by the temperature (Kaneko and Cahill, 2005; Vallone et al., 2005; López-Olmeda and Sánchez-Vázquez, 2011; Jerônimo et al., 2017). In developing larvae as well as zebrafish cell lines, temperature cycles of as little as 2°C difference, or temperature pulses, are able to entrain the circadian rhythms of clock genes (Vallone et al., 2005) and increase *per1, per2 and cry1, cry2* expression (Jerônimo et al., 2017). Endocrine factors are a mainstay of circadian biology. Their synthesis, secretion and also the sensitivity of the target organs to these signals are subject to a rigorous and often predictable control dependent on the time of day. Among the best-studied examples of endocrine factors that fluctuate over the course of the day are those whose production is governed by hypothalamic-pituitary axes, such as growth hormone (GH). In fish, Gh production is mainly stimulated by pituitary adenylate cyclase activating polypeptide (PACAP), which belongs to the same superfamily of neuropeptides as mammal GH-releasing hormone (GHRH) (Cowan et al., 2017). The current knowledge of fish physiology shows that their growth is dependent on photoperiod and temperature (Boeuf and Falcón, 2001). Growth-promoting effects are mostly mediated by IGFs (insulin-like growth factors), mainly IGF-1, which favor proliferation, differentiation, survival and cell migration (Butler and LeRoith, 2001; Reindl et al., 2011).

In addition, other hormones like glucocorticoids, vasopressin, adrenocorticotropic hormone, and thyrotropin exhibit pronounced circadian rhythms (Hastings et al., 2007). The endocrine and nervous systems respond together to a diversity of internal and external stimuli in fish. One of them is stress. It is worth mentioning that the stress caused by anthropological causes and environmental conditions (as temperature, food availability, climate seasonality, and photoperiod) affect the metabolism of the animal and generate adverse results such as development delay (Faught and Vijayan, 2016). In teleost fish, the major neuroendocrine circuit involved in stress response is the hypothalamic-pituitary-interrenal (HPI) axis. The cortisol is synthesized in the interrenal cells, and its release is part of the endocrine response evoked by stress, together with catecholamine and pro-opiomelanocortin secretion. Cortisol rhythms are endogenously driven by a circadian pacemaker, because they persist under constant environmental conditions (Auró de Ocampo and Ocampo, 1999; Cowan et al., 2017). Once in the cells, cortisol binds to the glucocorticoid receptor (GR) and ultimately modulates gene expression (Faught et al., 2016). The fish cortisol upregulates pathways involved in energy substrate mobilization, including gluconeogenesis, while downregulating energy demanding pathways, including growth and immune function (Faught and Vijayan, 2016). A glucocorticoid response element (GRE) was identified in the promoter of *Per1* gene suggesting a direct genomic action of glucocorticoids on the molecular clock components (Yamamoto et al., 2005).

In *D. rerio* and other teleost fish, besides the retina and peripheral tissues, the pineal gland cells are also capable of sensing the light (Davies et al., 2015) and are core components of the circadian system (Doyle and Menaker, 2007; Falcón et al., 2007). The pineal gland contains an intrinsic circadian clock that favors the rhythmic synthesis of melatonin. In teleosts, as well as other vertebrates, melatonin participates in the control of physiological process such as growth, food intake and digestion, reproduction, in addition to antioxidant activity and regulation of the immune system (Ngasainao and Lukram, 2016). Melatonin levels are higher in the dark due to light inhibition of the transcription and the stability of arylalkylamine *N*-acetyltransferase (*aanat*), an enzyme required for the hormone synthesis (Vatine et al., 2011). It is well established that melatonin counteracts stress-induced elevations of glucocorticoids directly on adrenal tissue, as reported in mammals and also suggested in fish, where peripheral (intraperitoneal) but not central (intracerebroventricular) melatonin injection is able to reduce cortisol secretion (Torres-Farfan et al., 2003; Azpeleta et al., 2010). Melatonin ability to reduce stress in teleosts has been usually associated with simultaneous changes in brain serotoninergic activity (Gesto et al., 2016; Sánchez-Vázquez et al., 2019). In addition, melatonin seems to modulate the GH production directly (on the pituitary gland) or indirectly (on the hypothalamus) (Boeuf and Falcón, 2001). After the teleost-specific whole-genome duplication, *D. rerio* has two distinct genes, *aanat1* and *aanat2*, respectively more expressed in the retina and the pineal gland (Vatine et al., 2011). Melatonin receptors also display a circadian rhythm (Quera and Hartley, 2012), and were identified as *mtnr1aa, mtnr1bb, mtnr1ab, mtnr1al*, and *mtnr1c* in *D. rerio*. It is well known that changes in temperature significantly affect the melatonin rhythm in ectothermic organisms (López-Olmeda and Sánchez-Vázquez, 2011).

Considering the effects of temperature in fish physiology, our aim was to investigate how temperature (23°C for 6 days), lower than the optimal (28°C), modulates expression of several gene pathways including growth hormone (*gh1*) and its receptors (*ghra, ghrb*), insulin-like growth factor 1 (*igf1a, igf1b*) and its receptors (*igf1ra, igf1rb*), cortisol and its receptor (*gr*), the limiting enzyme of melatonin synthesis (arylalkylamine N-acetyltransferase, *aanat*) and melatonin receptors (*mtnr1aa, mtnr1bb*), as well as their relationship with clock genes in *D. rerio* in early light and early dark phases of the day.

## Materials and Methods

### Animals

*Danio rerio* AB strain was bred in the zebrafish facility of the Department of Genetics and Evolutionary Biology in the Institute of Biosciences, University of São Paulo IB-USP, and donated to the Department of Physiology, IB-USP. Animals were maintained in glass tanks at 28 ± 1°C under photoperiod regimen of 14 h light and 10 hours dark (14:10 LD, lights on at 8:00 AM, ∼1000 lux) until reached ∼3 months of age. The fish were randomly fed three times a day with *Artemia* sp. (Artemia cyst, INVE, Salt Lake City, UT, United States, raised according to protocol established in the laboratory), in addition to commercial adult food (Zeigler^®^ Larval AP100, Gardners, PA, United States). The Central Vivarium of the Faculty of Medicine FM-USP (protocol 4172) carried out the sanitary control. The nomenclature used in this article follows the conventions established in: https://zfin.atlassian.net/wiki/spaces/general/pages/1818394635/ZFIN+Zebrafish+Nomenclature+Conventions.

### Experimental Protocol

**FIGURE 1:**
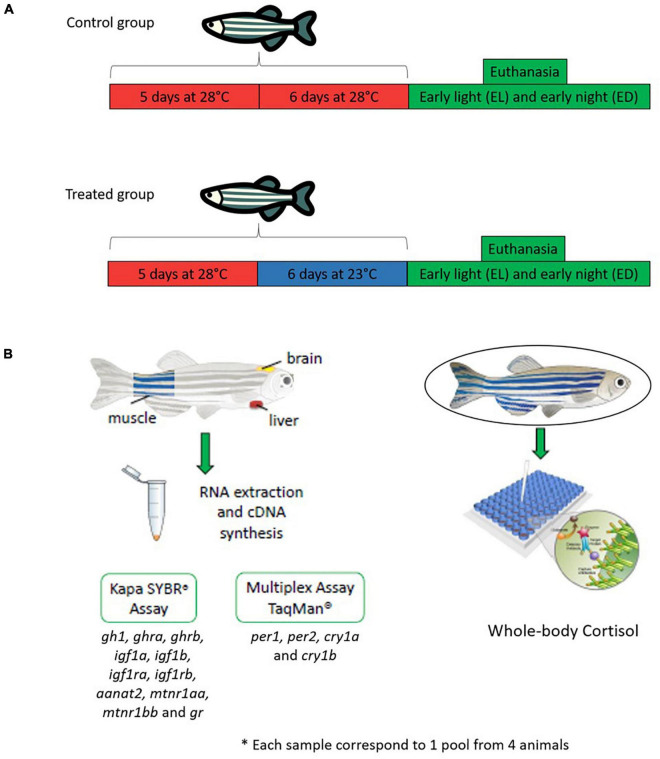
Experimental design. **(A)** *Danio rerio* male adults (*n* = 144) were divided in two large groups: a control temperature group (animals maintained at 28*^o^*C during 5 days and challenged 6 days at the same temperature) and a lower temperature group (animals maintained at 28*^o^*C during 5 days and challenged 6 days at lower temperature 23*^o^*C). After stimulation the animals were euthanized at early light (EL, 10 AM) and early dark (ED, 12 AM). **(B)** For the analysis, fish were separated into another two groups: a qPCR (96 animals) and a cortisol (48 animals) group. For the qPCR we evaluated *gh1, aanat2, mtnr1aa, mtnr1bb, opn4m1, opn4m2* in the brain, *ghra, ghrb, igf1a, igf1b, igf1ra, igf1rb, gr* in the liver and muscle. Also, in all tissues analyzed we performed a qPCR for clock genes (*per* and *cry).* Each experimental n comprised pooled tissues from four fish. The total number of samples used in each temperature and time point was *n* = 3–6.

This was a two-factor experimental design aiming to compare the responses to two temperatures (28 and 23*^o^*C) at two times of day [early light phase EL, 2 h after lights on, 10 AM, and early dark phase ED, 2 h after lights off, 12 AM (Figure 1)]. Adult males (*n* = 144, ∼0.150 g and ∼90 days old) were placed in a water recirculating system in two BOD (Bio-Oxygen Demand) incubators (Jobilnet, São Paulo, Brazil). Each incubator had a capacity for six 7 L-tanks (three tanks per temporal point), and a total water flow of 60 L. Physical (grid) and biological (Bio Filter Quartz Ceramic Ring, China) filters were used, and two thermostats were placed to ensure constant temperature. Each tank had a blue background according to Oliveira et al. (2015) and received light intensity of ∼1000 lux (white light, 420–750 nm) measured with the aid of a lux meter (LX-102, Lutron Electronic Enterprise, Taipei, Taiwan). The pH of the water was evaluated every 3 days using the LabconTest-pH-Tropical and adjusted, when necessary, with Labcon-Acid and or Labcon-Alcali (Alcon^®^, Camboriú, Santa Catarina, Brazil).

For the control group we placed and maintained 4 animals per tank for 11 days at 28°C (5 days of acclimation and 6 days of experimental period). For the treated group we also placed and maintained four animals per tank for 5 days (acclimation at 28°C) and then all the animals were transferred to an incubator at 23°C for 6 days (Figure 1A). At the end, the animals were euthanized by cryo-anesthesia (5 parts ice/1 part water, 0–4°C, during circa 30 s until the fish stopped its swimming movements), at EL (10 AM) and ED (12 AM). In order to avoid the postprandial peak of cortisol, the fish were not fed on the day of sampling. To obtain the brain, the head of the fish was removed and a dorsal cut was made from cranial to caudal, exposing the organ which was collected whole. To excise the liver, a ventral lengthwise cut exposed the abdominal organs, and the liver was then separated from the gastrointestinal tract. The muscle was excised from the caudal region of the fish by crosswise slicing, after isolating the skin and discarding the spine. The number of fish used for each experiment is described below and detailed in Supplementary Table S1. Each sample was a pool of four organs from four animals kept at each temperature and euthanized at two time points. Each experiment was independently repeated twice.

All procedures were performed according to the Ethics Committee on Use of Animals (CEUA) of the Institute of Biosciences, University of São Paulo, protocol N° 331/2018.

### Total RNA Extraction and Reverse Transcriptase Reaction

The quantitative polymerase chain reaction (qPCR) group consisted of 96 animals (*n* = 48 for each temperature). To obtain the samples, the brain, liver and muscle were removed from four fish and pooled. The final number of pooled samples was *n* = 3–6 for each temperature and temporal point (Figure 1B). The samples were stored at –80°C until processing, and then homogenized with sterile pestles in a final volume of 1 mL of Trizol (Ambion, Foster City, CA, United States). RNA extraction and purification were performed according to the manufacturer’s instruction. To prevent DNA contamination, each sample was treated with DNase I (turbo DNA-free™, Ambion, Foster City, CA, United States). The RNA concentration and quality were determined using a spectrophotometer (ND-1000 Spectrophotometer, NanoDrop, Wilmington, DE, United States). The cDNA synthesis was performed using 1 μg RNA, Superscript III, random hexamer primer and the reagents recommended by the manufacturer (ThermoFisher, Waltham, CA, United States). The samples containing the cDNA were stored at 4°C.

### Quantitative Polymerase Chain Reaction

As a reference gene for all organs 18S ribosomal RNA was chosen, tested, and validated for the stability of its expression in the various experimental conditions (data not shown). Housekeeping gene values were accepted as stables when the standard deviation (SD) < 1 across time points and temperature groups. The final concentrations of primers and probes were, respectively, 300 and 200 nM for the genes of interest, and 50 nM for primers and probe of 18S rRNA. In all assays, 50 ng of cDNA were used, and depending on the gene of interest, the samples were subject to multiplex reaction in two groups: *per1-cry1b* genes and *per2-cry1a* genes. In these reactions, KAPA PROBE FAST 2 mix (Kapa Biosystems, Wilmington, MA, United States) and the respective primers and probes were used. The remaining genes (Table 1) were individually evaluated in duplicates, using KAPA SYBR^®^ Fast qPCR Mix for iCycler^®^ 2x (Kapa Biosystems, United States). For the qPCR we evaluated *gh1, aanat2, mtnr1aa, mtnr1bb, opn4m1, opn4m2* in the brain, *ghra, ghrb, igf1a, igf1b, igf1ra, igf1rb, gr* in the liver and muscle. Also, in all tissues analyzed we performed a qPCR for clock genes (*per* and *cry*).

**TABLE 1:** Primers, probes, and access numbers for the qPCR assays.

Template	Sequence	Efficiency
18S RNA (X03205.1)	*Forward*: 5′-CGGCTACCACATCCAAGGAA-3′ *Reverse*: 5′-GCTGGAATTACCGCGGCT-3′ Probe:5′-/5TexRd-TGCTGGCACCAGACTTGCCCTC/BHQ_2/-3′	95%
*gh1* (NM_001020492)	*Forward*: 5′-ACAGCCTGACCATCGGAAAC-3′ *Reverse*: 5′-AATCCTCAAAAGGCAACGGC- 3′	98%
*ghra* (NM_001083578.1)	*Forward*: 5′-AGTCGTTCAGGGTTGCACTT-3′ *Reverse*: 5′-ACAGCGAACTCGCACTTCAT-3′	103%
*ghrb* (NM_001111081.2)	*Forward*: 5′-CGCTTAAGTGTGCCATGCTG-3′ *Reverse*: 5′-GGGCACATTCGAAGAAAGGC-3′	103%
*igf1a* (NM_131825)	*Forward: 5*′*-*CAGGCAAATCTCCACGATCTC-3′ *Reverse: 5*′*-*TTTGGTGTCCTGGGAATATCTGT-3′	114%
*igf1b* (NM_001115050)	*Forward: 5*′*-*GCAGCTCGTAGCGGTGGTCC-3′ *Reverse: 5*′*-*TCCACGCACACAACACTGGTCT-3′	105%
*igf1ra* (NM_152968)	*Forward*: 5′-ACCTGAGACCAGAGTGGCTA-3′ *Reverse*: 5′-TCTTTGGATCGGAGCGAGC-3′	106%
*igf1rb* (NM_152969)	*Forward*: 5′-AGGGTGGCCATTAAAACGGT-3′ *Reverse*: 5′-TTAATGGCGGCAAAGGCAAG-3′	104%
*aanat2* (NM_131411.2)	*Forward:* 5′-TGAAGACACCCATCAGCGTT-3′ *Reverse*: 5′-AGGACATTCACCAGACACCG-3′	110%
*mtrn1aa* (NM_131393.1)	*Forward*: 5′-TGGGAGTTCTCTGAACAGCTC-3′ *Reverse*: 5′-TTCCAGCCCCGGTGAAATATG-3′	108%
*mtrn1bb* (NM_131394.1)	*Forward*: 5′-TCCGGGATGCCAGAAAACATC-3′ *Reverse*: 5′-AGCGGGTATGGATAGAAAGCC-3′	110%
*per1* (NM_212439.2)	*Forward:* 5′-AGCTCAAACTCTCACAGCCCTT-3′ *Reverse:* 5′-TCAGAGCTGGCACTCAACAGA-3′ Probe: 5′-/5Cy5/TCCACCCAGCAGTTCTCTGGCATACA/BHQ_2/-3′	90–110% (Jerônimo et al., 2017; Sousa et al., 2017)
*per2* (NM_182857.2)	*Forward:* 5′-GTGGAGAAAGCGGGCAGC-3′ *Reverse:* 5′-GCTCTTGTTGCTGCTTTCAGTTCT-3′ Probe: - 5′-/6FAM/ATGGGTTCAGGATCAAACCGCTGT/BHQ_1/-3′	
*cry1a* (NM_001077297.2)	*Forward:* 5′-CTACAGGAAGGTCAAAAAGAACAGC-3′ *Reverse:* 5′-CTCCTCGAACACCTTCATGCC-3′ Probe: 5′-/5HEX/AAAGCGTGGGTTGTTTGTAGCAGC/BHQ_1/-3′	
*cry1b* (NM_131790.4)	*Forward*: 5′-CGTCTCTGGAGGAGCTCGG-3′ *Reverse*: 5′-TCTCCCCCGGGCCAC-3′ Probe: 5′/5HEX/TTTGAAACAGAGGGACTGTCCACTGCTG/BHQ_1/-3′	
*gr* (NM_001020711.3)	*Forward*: 5′-GAGGAGAACTCCAGCCAGAAC- 3′ *Reverse:* 5′-TTCACAAAGGTGTAGAAGCAGAAG-3′ Probe: 5′-/5HEX/AGGAGTCCACCCACCAAGTCGTGC/BHQ_1/-3′	

*Nomenclature of Danio rerio genes and proteins follows https://zfin.atlassian.net/wiki/spaces/general/pages/1818394635/ZFIN+Zebrafish+Nomenclature+Conventions.*

Primers and probes were designed with the PrimerBlast program or Integrated DNA Technologies (IDT, Coralville, IA, United States), according to GenBank^1^ sequences (Table 1), and synthesized by ThermoFisher (Waltham, CA, United States) (hormone receptor genes) or IDT (clock and *gh1* gene). Primers’ efficiencies were determined using serial dilutions (1, 1:2, 1:4, 1:8, and 1:16) of single cDNA samples. The efficiency for each primer pair was calculated according to the equation: 10^∧^ (−1/*x*)−1(100), in which *x* corresponds to the slope of the linear regression curve. Values between 90 and 110% were considered as indicators of appropriate efficiency.

All assays were performed in a thermocycler iQ5 (BioRad Laboratories, Hercules, CA, United States) under the following conditions: SYBR^®^ GreenER™ – 10 min at 95°C, followed by 45 cycles of 15 s at 95°C, 1 min at 60°C and 80 cycles of 10 s at 55°C, with gradual increase of 0.5°C; for the multiplex reaction – 3 min at 95°C followed by 45 cycles of 15 s at 95°C and 60 s at 60°C.

Gene expression was quantified according to the 2^–Δ^ ^Δ^ *^CT^* method (Livak and Schmittgen, 2001). Cycle threshold (CT) for each reaction was determined as the number of cycles of the geometric portions of the amplification curves crossed by the threshold line. The ΔCT was determined by subtracting the 18S RNA CT from the CT of the gene of interest at the same time point and temperature, both corresponding to the average of replicates of the same cDNA sample. The mean value obtained from control temperature (28°C) at EL was subtracted from all other values, obtaining the ΔΔCT, which was used as the negative exponential of base 2 (2^–Δ^ ^Δ^ *^CT^*). Results were expressed as mean ± standard error of the mean (SEM), and fold change was calculated as transcripts of A/transcripts of B between experimental conditions.

### Quantitative Determination of Total Cortisol (ELISA Kit)

The procedure of cortisol extraction was adapted from Alderman and Bernier (2009) and Egan et al. (2009). The cortisol group consisted of 48 animals (*n* = 24 for each temperature). To obtain the samples, the animals were euthanized at EL and ED, weighed, and placed in 2 mL plastic tubes. Each sample corresponded to a pool of four animals. The final number of pooled samples was *n* = 3 for each temperature and temporal point (Figure 1B). The samples were homogenized in 500 μL of ice-cold 1X PBS buffer using an X1000 Homogenizer Drive (CAT Scientific, Paso Robles, CA, United States), and processed according to the kit instructions (Salivary Cortisol ELISA kit, Salimetrics, Carlsbad, CA, United States). The optical densities (OD) for the duplicate wells were averaged, the non-specific binding OD was subtracted from all wells, and the percent bound was calculated dividing the mean OD of each duplicate (B) by the mean of zero (Bo). The whole-body cortisol concentrations were interpolated in the standard curve using a 4-parameter non-linear regression curve fit (if *r* > 0.99), normalized with the weight of each sample (each one a pool of four animals) and reported as cortisol concentrations (ng/g body weight) (according to Egan et al., 2009). Results were expressed as mean ± standard error of the mean (SEM).

### Statistical Analysis

The log values of qPCR and the hormone concentrations, in the control temperature (28*^o^*C) and treated (23*^o^*C) groups, in both time points, were tested for normality and compared to each other by two-way ANOVA. To evaluate the interaction between factors (time point and temperature) the *F* value was analyzed [*F*(DFn, DFd), *p* < 0.05], followed by a Bonferroni’s test to compare the temporal points or temperatures factor, considering significant differences when *p* < 0.05.

For the correlation analysis, the log values of qPCR (2^–Δ^ ^Δ^ *^CT^*) were analyzed by Pearson correlation coefficients, with a multiple comparison test, and significance set for *p* < 0.05. The correlation coefficient, *r*, ranges from –1 to +1, assuming + 1 as perfect correlation, 0–0.99 the two variables tend to increase or decrease together, 0 the two variables do not vary together at all, 0 to –0.99 one variable increases as the other decreases and –1 as perfect negative or inverse correlation. All analyses were carried out in GraphPad Prism Version 7.0 (La Jolla, CA, United States).

## Results

### Growth Hormone Axis

Considering that fish growth is affected by the photoperiod and temperature (Boeuf and Falcón, 2001), we initially investigated the effect of lower temperature as a cold stressor on the GH axis. At molecular level we evaluated the expression of the growth hormone gene (*gh1)* in the brain, its receptors (*ghra and ghrb*) and the insulin-like growth factor 1 (*ig1a and igf1b*) and its receptors (*ig1ra and igf1rb*) in the liver and muscle.

**FIGURE 2:**
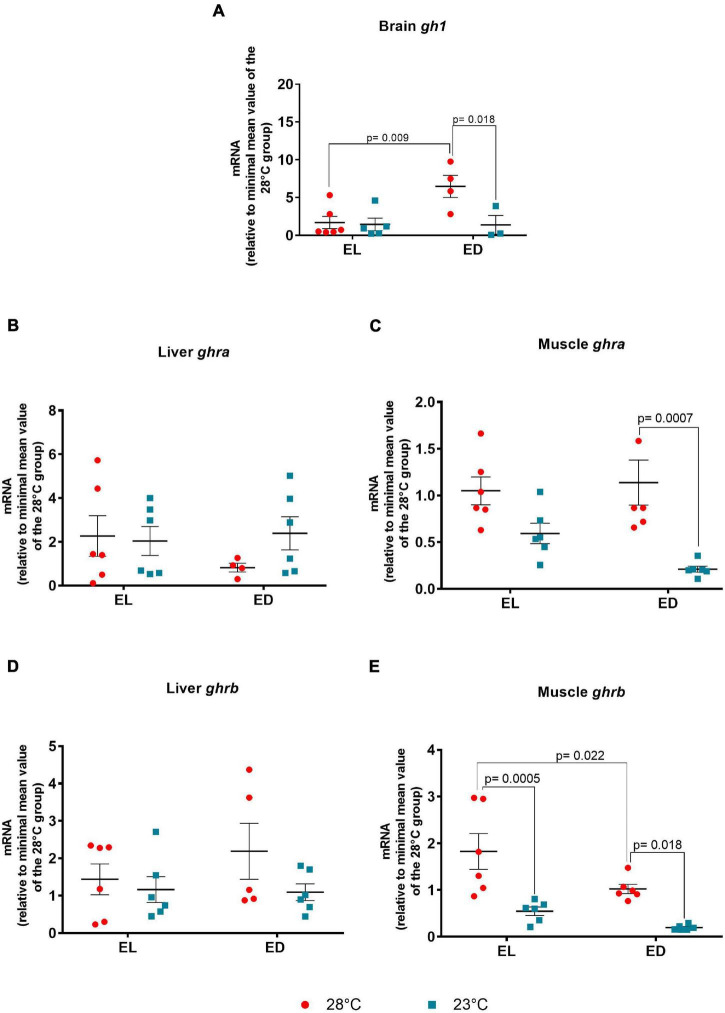
Early light (EL) and early dark (ED) gene expression of growth hormone (*gh1*) and growth hormone receptors (*ghra* and *ghrb*) in adult *Danio rerio*. **(A)** Brain *gh1*; **(B)** liver *ghra*; **(C)** muscle *ghra*; **(D)** liver *ghrb*; **(E)** muscle *ghrb*. The gene expression was normalized by the 18S rRNA and expressed relative to the lowest mean of the control temperature group. The values are expressed as mean ± SEM. *n* = 3–6 (pools of four animals each). Significant differences are shown when *p* < 0.05. Red symbol corresponds to 28*^o^*C and blue symbol corresponds to 23*^o^*C.

We found a significant interaction between time and temperature of *gh1* [*F*(1,14) = 5.068, *p* = 0.04] in the brain. It should be noticed that the *gh1* gene expression was higher (fourfold change, *p* = 0.009) at early dark phase (ED) in comparison to early light phase (EL) in fish subjected to 28°C (Figure 2A). A reduction of expression (fivefold change, *p* = 0.018) was found in the lower temperature-treated animals at ED as compared to EL (Figure 2A). Due to this decrease of mRNA level, the difference seen between the analyzed time points was abolished, due to dampening of the amplitude or shift of the expression peak (Figure 2A).

In the liver, the GH receptor *ghra* did not vary between EL and ED at 28 or 23*^o^*C (Figure 2B). In the muscle, *ghra* (Figure 2C) did not vary between time points at both temperatures, but its transcript exhibited a marked reduction (fivefold change, *p* = 0.0007) in 23°C-exposed animals at ED compared to the control temperature group at the same time of day. The *ghrb* expression showed no variation in the liver of animals from both temperatures (Figure 2D). On the other hand, the *F* value of muscle *ghrb* indicated a significant impact for each stimulus (mostly temperature) [time point *F*(1,20) = 8.078, *p* = 0.01], [temperature *F*(1,20) = 26.91, *p* < 0.0001] but no interaction between them. The *ghrb* mRNA level in the muscle displayed a decrease (twofold change, *p* = 0.022) at ED compared to EL in the animals at 28*^o^*C and a reduction at the lower temperature at EL (threefold change, *p* = 0.0005) and ED (fivefold change, *p* = 0.018), as compared to the control temperature group (Figure 2E).

**FIGURE 3:**
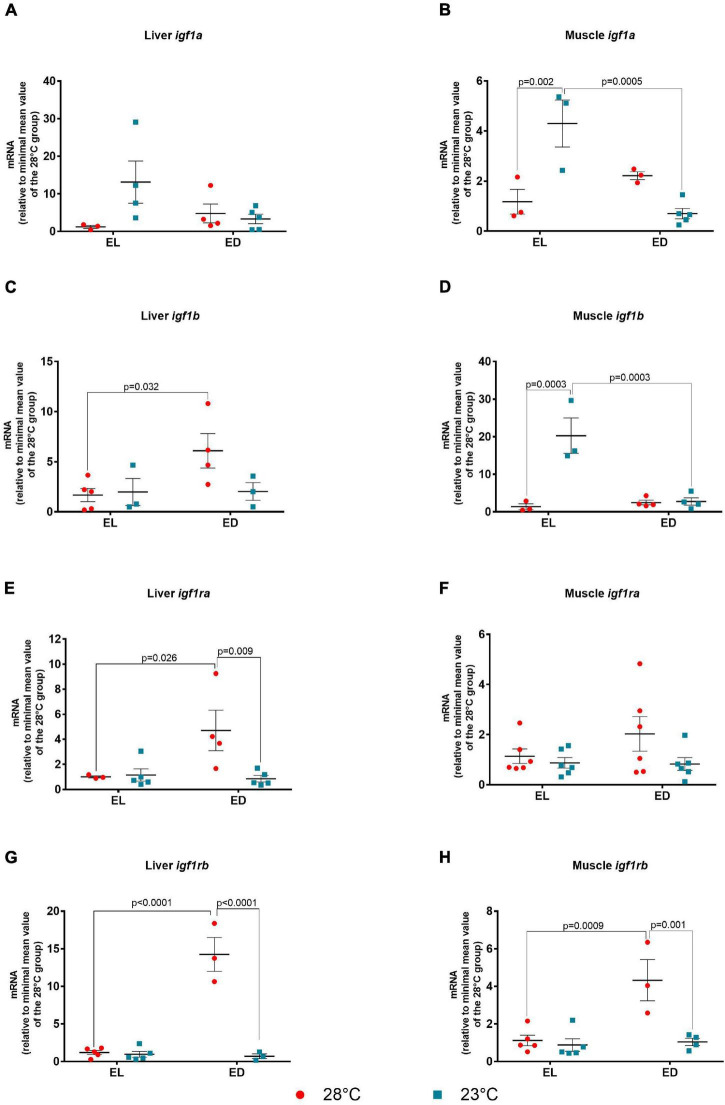
Early light (EL) and early dark (ED) gene expression of insulin-like factors (*igf1a* and *igf1b*), and their receptors (*igf1ra* and *igf1rb*) in adult *Danio rerio*. **(A)** Liver *igf1a*; **(B)** muscle *igf1a*; **(C)** liver *igf1b*; **(D)** muscle *igf1b*; **(E)** liver *igf1ra*; **(F)** muscle *igf1ra*; **(G)** liver *igf1rb*; **(H)** muscle *igf1rb.* The gene expression was normalized by the 18S rRNA and expressed relative to the lowest mean of the control temperature group. The values are expressed as mean ± SEM. *n* = 3–6 (pools of four animals each). Significant differences are shown when *p* < 0.05. Red symbol corresponds to 28*^o^*C and blue symbol corresponds to 23*^o^*C.

Next, we evaluated other components of the GH pathway such as the insulin-like growth factors *igf1a* and *igf1b* and their receptors, *igf1ra* and *igf1rb*. The expression of *igf1a* in the liver showed no statistical differences between temperatures or times of day (*F* value not significant) (Figure 3A). However, the ANOVA analysis for the muscle *igf1a* showed significant interaction between the two factors [*F*(1,10) = 23.23, *p* = 0.0007]. Also in the muscle, there was no difference between the expression at EL and ED at 28*^o^*C, but a reduction at ED compared to EL at 23*^o^*C was observed (sixfold change, *p* = 0.0005) (Figure 3B). Still in the muscle, an increase in *igf1a* mRNA was observed at 23*^o^*C in comparison to 28*^o^*C at EL (fourfold change, *p* = 0.002). In the liver, the F value was not significant for the interaction between time and temperature. However, the *igf1b* transcripts showed the highest expression at ED compared to EL at 28°C suggesting a nocturnal peak (fourfold change, *p* = 0.032); no statistical differences were found between EL and ED at 23*^o^*C (Figure 3C). The *F* value for the muscle *igf1b* indicated significant interaction between time and temperature [*F*(1,10) = 19.48, *p* = 0.001]. The muscle *igf1b* transcripts were found to decrease at ED compared to EL at 23*^o^*C (sevenfold change, *p* = 0.0003) and to increase at 23*^o^*C compared to 28*^o^*C at EL (14-fold change, *p* = 0.0003) (Figure 3D).

As to *Igf* receptors, the ANOVA analysis for the hepatic *igf1ra* showed significant interaction between the factors [*F*(1,13) = 5.683, *p* = 0.03]. In this organ, the *igf1ra* showed the highest expression at ED compared to EL at 28°C (fivefold change, *p* = 0.026), and a decrease at 23*^o^*C at ED when compared to 28°C (fivefold change, *p* = 0.009) (Figure 3E). There were no differences in the muscle *igf1ra* between time points or temperatures (*F* value not significant) (Figure 3F).

The *F* value of hepatic *igf1rb* showed a significant interaction between time and temperature [*F*(1,12) = 56.37, *p* < 0.0001]. The *igf1rb* transcripts in the liver exhibited similar profile to *igf1ra.* An increase at 28*^o^*C at ED compared to EL (12-fold change, *p* < 0.0001) and a marked decrease at 23*^o^*C compared to 28*^o^*C at ED (20-fold change, *p* < 0.0001) (Figure 3G). In the muscle, similar differences between time points (fourfold change, *p* = 0.0009) and temperatures (fourfold change, *p* = 0.001) were observed (Figure 3H). The *F* value showed a significant interaction between both factors [*F*(1,13) = 10.67, *p* = 0.006].

### Cortisol Axis

Another hormone involved in teleost growth is cortisol which decreases muscle protein and hepatic glycogen levels and induces hyperglycemia (Auró de Ocampo and Ocampo, 1999; Baltzegar et al., 2015).

**FIGURE 4:**
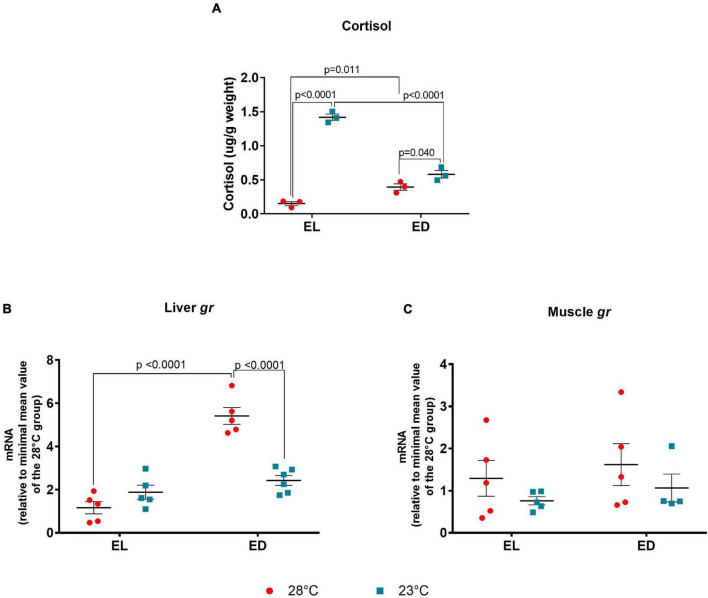
Early light (EL) and early dark (ED) whole body cortisol and gene expression of glucocorticoid receptor (*gr*) in adult *Danio rerio*. **(A)** Cortisol levels; **(B)** liver *gr*; **(C)** muscle *gr*. The hormone concentration (μg) was normalized by sample weight (g, Supplementary Table S2). The gene expression was normalized by the 18S rRNA and expressed relative to the lowest mean of the control temperature group. The values are expressed as mean ± SEM. *n* = 3 (pools of four animals each). Significant differences are shown when *p* < 0.05. Red symbol corresponds to 28*^o^*C and blue symbol corresponds to 23*^o^*C.

Since temperature changes (–5*^o^*C) are cold stressful factor, we analyzed cortisol concentration in the whole-body extract (animals’ weights are shown in Supplementary Table S2). The ANOVA analysis showed a significant interaction between the two factors [*F*(1,8) = 140.9, *p* < 0.0001]. We found an increase in the cortisol levels at ED compared to EL in the control temperature group (*p* = 0.011) and the opposite effect at 23*^o^*C (*p* < 0.0001) (Figure 4A). Unexpectedly, the control temperature group did not show higher levels in the light phase possibly because its peak preceded the time of sample collection. However, we observed an increase in cortisol (μg/g body weight) in animals at 23°C in the EL (*p* < 0.0001) and ED (*p* = 0.040) as compared to the control temperature group at the same time points (Figure 4A).

We next analyzed the glucocorticoid receptor (*gr*) expression in metabolic tissues as liver and muscle. In the liver, the ANOVA analysis indicated significant interaction between time of day and temperature [*F*(1,17) = 36.64, *p* < 0.0001]. In addition, we found a significant peak in *gr* expression at ED in animals at 28*^o^*C (Figure 4B) (fivefold change, *p* < 0.0001); when subject to 23*^o^*C, there were no differences between EL and ED. At ED, the transcripts of *gr* decreased at 23*^o^*C (twofold change, *p* < 0.0001) as compared to 28*^o^*C (Figure 4B). In the muscle, the *gr* expression showed no significant differences between day and night at the same temperature or between temperatures at the same time point (Figure 4C).

### Melatonin Axis

Based on the interactions of melatonin with the previously evaluated hormone axis, we decided to investigate whether this pathway (at the molecular level) was affected by temperature variation, analyzing the gene expression of the enzyme arylalkylamine *N*-acetyltransferase and melatonin receptors.

**FIGURE 5:**
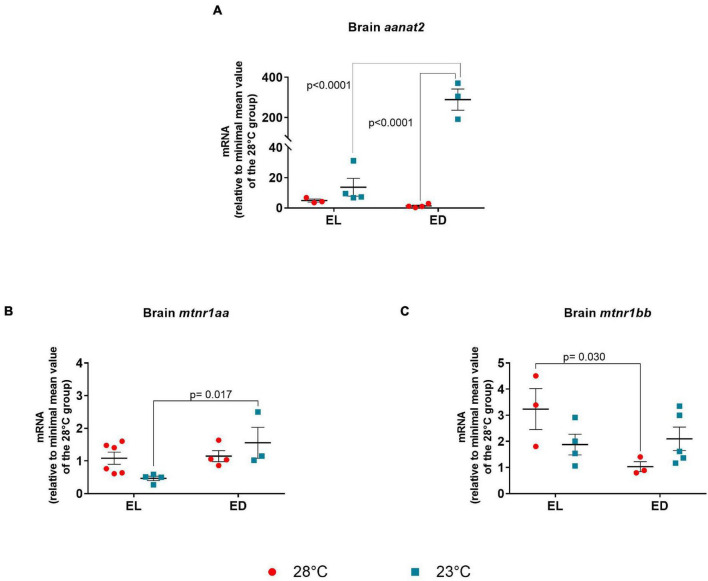
Early light (EL) and early dark (ED) gene expression of arylalkylamine *N*-acetyltransferase (*aanat2*) and melatonin receptors (*mtnr1aa* and *mtnr1bb*) in adult *Danio rerio*. **(A)** Brain *aanat2*; **(B)** Brain *mtnr1aa*; **(C)** brain *mtnr1bb.* The gene expression was normalized by the 18S rRNA and expressed relative to the lowest mean of the control temperature group. The values are expressed as mean ± SEM. *n* = 3–6 (pools of four animals each). Significant differences are shown when *p* < 0.05. Red symbol corresponds to 28*^o^*C and blue symbol corresponds to 23*^o^*C.

In the brain, *aanat2* expression was evaluated. The ANOVA analysis showed significant interaction between time and temperature [*F*(1,10) = 39.75, *p* < 0.0001]. This gene transcripts showed no difference between time points at 28°C but showed an increase at ED compared to EL at 23*^o^*C (21-fold change, *p* < 0.0001) (Figure 5A). On the other hand, a remarkable increase of *aanat2* mRNA was induced in animals exposed to 23°C when compared with the control temperature animals at 28°C, at ED (214-fold change, *p* < 0.0001) (Figure 5A). As expected, *aanat2* expression peaked in the dark at 23*^o^*C which may suggest an increase of melatonin synthesis in lower temperature-exposed animals.

Melatonin plays a key role in several tissues, but we focused on the hormone central sources and its local effects. In the brain, the melatonin receptors *mtnr1aa* and *mtnr1bb* were evaluated. The ANOVA analysis showed a significant interaction between the two factors for both genes [*mtnr1aa F*(1,13) = 4.896, *p* = 0.04] and [*mtnr1bb F*(1,11) = 5.923, *p* = 0.03]. Temporal variation was seen for *mtnr1aa* mRNA in the brain at 23*^o^*C, with peak in the ED phase (threefold increase, *p* = 0.017) (Figure 5B). The transcripts of *mtnr1bb* showed a decrease at ED compared to EL in the control temperature group (threefold decrease, *p* = 0.030) (Figure 5C).

### Clock Genes

Finally, we investigated if a change of 5°C would be sufficient to affect the molecular clock in adult *D. rerio*. Previous results from literature and from our laboratory indicated that clock genes such as *clock* (in *D. rerio* larvae at 20 and 30*^o^*C) and *bmal* (in *D. rerio* ZEM-2S cells at 28 and 33*^o^*C) are not affected by temperature changes (Lahiri et al., 2005; Jerônimo et al., 2017), so we chose to evaluate the negative clock genes, *per* and *cry* in all tissues evaluated.

**FIGURE 6:**
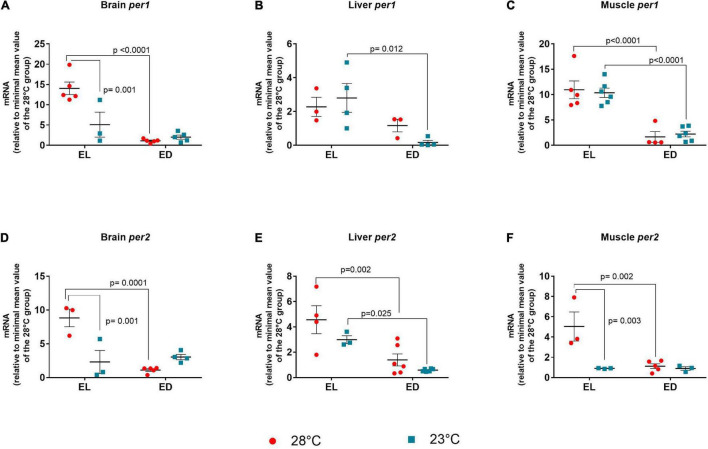
Early light (EL) and early dark (ED) expression of period (*per1* and *per2*) genes in adult *Danio rerio*. **(A)** Brain *per1*; **(B)** liver *per1*; **(C)** muscle *per1*; **(D)** brain *per2*; **(E)** liver *per2*; **(F)** muscle *per2.* The gene expression was normalized by the 18S rRNA and expressed relative to the lowest mean of the control temperature group. The values are expressed as mean ± SEM. *n* = 3–6 (pools of four animals each). Significant differences are shown when *p* < 0.05. Red symbol corresponds to 28*^o^*C and blue symbol corresponds to 23*^o^*C.

The ANOVA analysis for *per1* indicated a significant interaction between the two factors only in the brain [*F*(1,14) = 13.17, *p* = 0.002]. In the liver and muscle an increase of *per1* expression was observed in EL animals as compared to ED fish [liver *F*(1,10) = 10.33, *p* = 0.009], [muscle *F*(1,17) = 59.92, *p* < 0.0001]. At 28°C, *per1* had higher expression at EL compared to ED in the brain (13-fold change, *p* < 0.0001) and muscle (sevenfold change, *p* < 0.0001), evidencing that the gene peaks in the light phase (Figures 6A–C). In the brain, there was a decrease in *per1* expression at 23°C at EL as compared to the control temperature animals (threefold decrease, *p* = 0.001), which demonstrate that low temperatures disrupt (reduced or phase shifted) *per1* expression (Figure 6A). At 23°C, we also observed a decrease in *per1* expression at ED compared to EL in the liver (17-fold decrease, *p* = 0.012) and the muscle (fivefold decrease, *p* < 0.0001) (Figures 6B,C).

The ANOVA analysis of *per2* showed significant interaction between factors in the brain [*F*(1,11) = 23.16, *p* = 0.0005] and the muscle [*F*(1,10) = 9.258, *p* = 0.01]. In the liver a higher expression of *per2* was seen in EL [*F*(1,15) = 23.26, *p* = 0.0002]. Like *per1*, *per2* mRNA in the brain (Figure 6D), liver (Figure 6E), and muscle (Figure 6F) of animals kept at 28°C exhibited higher expression at EL than at ED (*p* = 0.0010; *p* = 0.002; *p* = 0.002, respectively), peaking in the early light phase. This peak was also observed only in the liver at 23*^o^*C (*p* = 0.025) (Figure 6E).

In the brain and muscle, there was a significant decrease in gene expression of *per2* in EL in animals kept at 23°C compared to the control temperature group (brain, fourfold decrease, *p* = 0.001), (muscle, fivefold decrease, *p* = 0.003) (Figures 6D,F).

**FIGURE 7:**
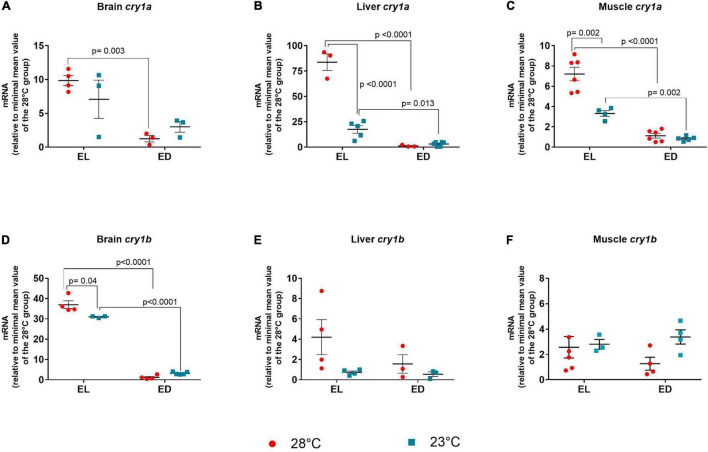
Early light (EL) and early dark (ED) expression of cryptochrome (*cry1a* and *cry1b*) genes in adult *Danio rerio*. **(A)** Brain *cry1a*; **(B)** liver *cry1a*; **(C)** muscle *cry1a*; **(D)** brain *cry1b*; **(E)** liver *cry1b*; **(F)** muscle *cry1b.* The gene expression was normalized by the 18S rRNA and expressed relative to the lowest mean of the control temperature group. The values are expressed as mean ± SEM. *n* = 3–6 (pools of four animals each). Significant differences are shown when *p* < 0.05. Red symbol corresponds to 28*^o^*C and blue symbol corresponds to 23*^o^*C.

For *cry1a*, a significant interaction between the two factors was found in the liver [*F*(1,13) = 83.54, *p* < 0.0001] and the muscle [*F*(1,17) = 17.63, *p* = 0.0006)]. In the brain a higher transcript level was demonstrated in the EL group [*F*(1,9) = 19.4, *p* = 0.0017]. In all three organs, the expression of *cry1a* at 28°C at EL was significantly higher than at ED (brain *p* = 0.003; liver *p* < 0.0001; muscle *p* < 0.0001, respectively) (Figures 7A–C). This light phase peak was evidenced also in the liver (*p* = 0.013) and muscle (*p* = 0.002) at 23*^o^*C. In the liver (Figure 7B) and muscle (Figure 7C), although the expression peak of *crya* was still maintained at the lower temperature, its amplitude was significantly decreased (liver, fivefold decrease, *p* < 0.0001), (muscle, twofold decrease, *p* = 0.002).

When the *F* value of *cry1b* was analyzed, a significant interaction between the two factors was seen only in the brain [*F*(1,12) = 14.72, *p* = 0.002]. The expression in the brain of animals kept at 28°C or 23*^o^*C peaked in the EL with a considerable reduction at ED (30-fold decrease at 28*^o^*C and ninefold decrease at 23*^o^*C, *p* < 0.0001) (Figure 7D). Also, the brain showed decreased *cry1b* expression in the EL at lower temperature (1.2-fold decrease, *p* = 0.004) as compared to the control temperature group, but the peak was still observed in EL. Liver and muscle *cry1b* transcripts showed no statistical differences between time points or temperatures (Figures 7E,F).

In summary, regardless of the temperature a general pattern of transcripts was seen in the organs evaluated, which exhibited higher clock gene expression during the day (Table 2).

**TABLE 2:** Overview of temperature or time point effect on hormonal pathways.

Gene/Cortisol	Organ	Alterations in gene expression and cortisol levels
*gh1*	Brain	Decrease in the treated animals (23*^o^*C) compared to control (28*^o^*C) at ED. Increase in the control group (28*^o^*C) at ED compared to EL.
*ghra*	Muscle	Decrease in the treated animals (23*^o^*C) compared to control (28*^o^*C) at ED.
*ghrb*	Muscle	Decrease in the treated animals (23*^o^*C) compared to control (28*^o^*C) at ED and EL. Decrease in the control group (28*^o^*C) at ED compared to EL.
*igf1a*	Muscle	Increase in the treated animals (23*^o^*C) compared to control (28*^o^*C) at EL. Decrease in the treated group (23*^o^*C) at ED compared to EL.
*igf1ra*	Liver	Decrease in the treated animals (23*^o^*C) compared to control (28*^o^*C) at ED. Increase in the control group (28*^o^*C) at ED compared to EL.
*igf1b*	Liver	Increase in the control group (28*^o^*C) at ED compared to EL.
	Muscle	Increase in the treated animals (23*^o^*C) compared to control (28*^o^*C) at EL. Decrease in the treated group (23*^o^*C) at ED compared to EL.
*igf1rb*	Liver	Decrease in the treated animals (23*^o^*C) compared to control (28*^o^*C) at ED. Increase in the control group (28*^o^*C) at ED compared to EL.
	Muscle	Decrease in the treated animals (23*^o^*C) compared to control (28*^o^*C) at ED Increase in the control group (28*^o^*C) at ED compared to EL.
cortisol	Whole-body	Increase in the treated animals (23*^o^*C) compared to control (28*^o^*C) at ED and EL. Decrease in the treated group (23*^o^*C) at ED compared to EL.
*gr*	Liver	Decrease in the treated animals (23*^o^*C) compared to control (28*^o^*C) at ED. Increase in the control group (28*^o^*C) at ED compared to EL.
*aanat2*	Brain	Increase in the treated animals (23*^o^*C) compared to control (28*^o^*C) at ED. Increase in the treated group (23*^o^*C) at ED compared to EL.
*mtnr1aa*		Increase in the treated group (23*^o^*C) at ED compared to EL.
*mtnr1bb*		Decrease in the control group (28*^o^*C) at ED compared to EL.
*per1*	Brain	Decrease in the treated animals (23*^o^*C) compared to control (28*^o^*C) at EL. Decrease in the control group (28*^o^*C) at ED compared to EL.
	Liver	Decrease in the treated group (23*^o^*C) at ED compared to EL.
	Muscle	Decrease in both control (28*^o^*C) and treated (23*^o^*C) groups at ED compared to EL.
*per2*	Brain	Decrease in the treated animals (23*^o^*C) compared to control (28*^o^*C) at EL. Decrease in the control group (28*^o^*C) at ED compared to EL.
	Liver	Decrease in both control (28*^o^*C) and treated (23*^o^*C) groups at ED compared to EL.
	Muscle	Decrease in the treated animals (23*^o^*C) compared to control (28*^o^*C) at EL. Decrease in the control group (28*^o^*C) at ED compared to EL.
*cry1a*	Brain	Decrease in the control group (28*^o^*C) at ED compared to EL.
	Liver	Decrease in the treated animals (23*^o^*C) compared to control (28*^o^*C) at EL.
	Muscle	Decrease in both control (28*^o^*C) and treated (23*^o^*C) groups at ED compared to EL.
*cry1b*	Brain	

### Correlation Analysis

**FIGURE 8:**
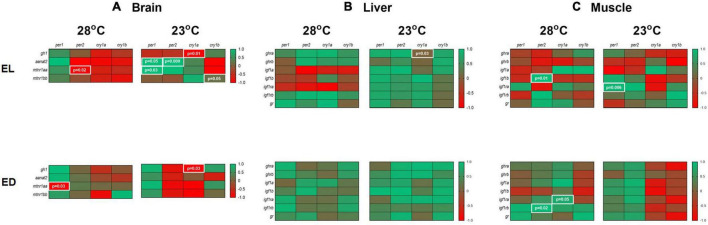
Correlation of gene expression in the brain, liver and muscle of adult *Danio rerio*. **(A)** Brain. Four positive and four negative correlations; **(B)** liver. One positive correlation; **(C)** muscle. Four positive correlations. Data from the same sample, temperature and time point with a Gaussian distribution were analyzed by Pearson correlation coefficients. The values are expressed as coefficient *r*. Values: 1 perfect correlation; 0 to 0.99 the two variables tend to increase or decrease together; 0 the two variables do not vary together at all; 0 to –0.99 one variable increases as the other decreases and –1 as perfect negative or inverse correlation. Significance was set for *p* < 0.05, shown in white letters inside white boxes.

Once the gene expression values were obtained, we performed a Pearson correlation analysis between the expression of clock genes and every other gene in the different tissues. It should be noted that this type of analysis offers a hypothetical relationship that cannot necessarily be evidenced in the physiology of the animal. A heat map was built to present the results: samples correspond to the same day time (EL or ED) and the temperature conditions (28 or 23°C) (*p* < 0.05) (Figure 8).

In the brain, at 28*^o^*C, we observed negative correlations between *per1/mtnr1aa* and *per2/mtnr1aa.* At 23°C, we found positive correlations for *per1/aanat2, per1/mtnr1aa, per2/aanat2* and *cry1b/mtnr1bb.* A negative correlation was found in the cold-exposed group between *cry1a/gh1* (*p* < 0.05) (Figure 8A).

In the liver, only a positive correlation was observed between *cry1a/ghra* at 23°C group (*p* < 0.05) (Figure 8B). In the muscle, at 28°C, positive correlations were observed between *per2/igf1b, per2/igf1rb* and *cry1a/igf1ra* (*p* < 0.05) (Figure 8C). At 23°C, a positive correlation between *per1/igf1ra* (*p* < 0.05) was seen (Figure 8C).

**FIGURE 9:**
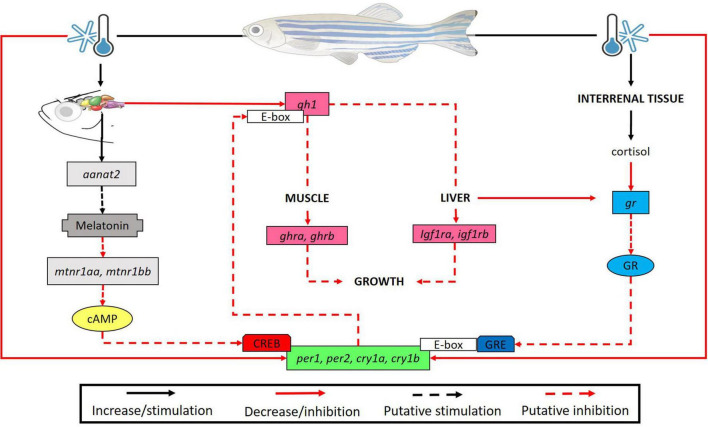
Brain of *D. rerio* submitted to low temperatures show high levels of the *aanat2* transcripts causing a putative increase in melatonin synthesis. We suggested that expression of melatonin receptors (*mtnr1aa* and *mtnr1bb*) may decrease to ensure homeostasis. In turn, it is known that *mtnr1aa, mtnr1bb* (Gi protein-coupled receptors) inhibit adenylate cyclase activity which ultimately leads to the inhibition of CREB phosphorylation, a transcription factor required for clock gene expression, resulting in a reduction of their transcripts. Also, in the brain we found a reduction in the *gh1* transcripts at lower temperature, leading to a decrease of *ghra* and *ghrb* in the muscle, and *igf1ra, igf1rb* in the liver, suggesting a long-term inhibition of the animal growth. In turn, clock genes are capable to bind to the promoter E-box region of genes as *gh1* thus modulating its expression (a putative inhibition in our study). On the other hand, there was an increase in the cortisol concentration and a compensatory reduction of its receptor transcripts. The glucocorticoid (Gc)/glucocorticoid receptor complex modulates the expression of clock genes by binding to glucocorticoid response elements (GRE) in their promoters. Consequently, we evidenced a decrease in the expression of clock genes in all organs analyzed. This may be associated to cold-modulation by melatonin and cortisol pathways or a direct effect of low temperature on clock gene transcripts.

Considering these data, we suggest an interaction (positive or negative) between clock genes and a few genes of the hormonal pathways studied including those of the growth and melatonin pathways (Figure 9).

## Discussion

In several teleost species, GH secretion is increased at higher temperatures suggesting accelerated growth during spring/summer and reduced GH levels in the winter (Deane and Woo, 2009). The otoliths commonly known as “ear stones” are hard, calcium carbonate structures, located directly behind the teleost brain. Its measurement has frequently been used as a proxy for somatic growth in marine and freshwater fish species (Rudy and Forsberg, 2016). In *D. rerio* and other teleosts, the environmental temperature modulates the growth of the otoliths due to an up- downregulation of the GH axis (Wang et al., 2015; Grønkjær, 2016; Loeppky et al., 2021). When *gh1* in the brain was evaluated, we showed that gene expression peaked in the early dark in control temperature animals with a notable reduction in animals kept at lower temperature, demonstrating the nocturnal pattern of this hormone. Also, according to Cowan et al. (2017) the highest GH production usually coincides with the highest water temperature in several fish species. In fish, the cAMP signaling pathway leads to increased *gh1* mRNA transcription and hormone release through the activation of transcription factors such as *c-fos* (Klein and Sheridan, 2008). In mammals, the cryptochrome (*Cry*) genes are known to inhibit the accumulation of cAMP by interacting with Gs protein (Zhang et al., 2010). According to the correlation analyses, we demonstrate this modulation by the clock genes (specifically *per1* and *cry1a*) on *gh1* transcripts. In this way, the *gh1* is down-regulated not only by the lower temperature as by the time of the day in *D. rerio* adults.

Due to their ectothermic physiology, tropical fish in cold waters decrease their metabolism, resulting in a reduction of growth, probably due to a decrease of both GH production and the affinity and/or number of its receptors (White et al., 2012). No difference in size and weight was found in animals from the two temperatures, probably due to the short (6 days) exposure to 23*^o^*C (data not shown). Molecular alterations probably occur before those changes reflect as actual growth. Our results agree with what was reported by Gabillard et al. (2006) who kept rainbow trout (*Oncorhynchus mykiss*) at two temperatures (12 and 4°C) and obtained an increase for *ghra* transcripts when the water temperature was raised and a remarkable decrease at low temperature.

The liver is the major source of circulating IGF1 in fish (Leroith et al., 2001). In our study, we evidenced high levels specifically of the hepatic *igf1b* in the temperature control group at ED, corresponding with the *gh1* peak at the same time of the day. Curiously, the muscle presented an increase of *igf1a* and *igf1b* in the lower temperature group during EL, so we hypothesized that in this organ the protein is acting locally to compensate the cold-induced down-regulation of the GH axis. In agreement with our study, in zebrafish larvae exposed to low temperatures the *igfbp1a* (or *igf1a*) expression was up-regulated (Long et al., 2013).

The *igf1ra/igf1rb* in the liver showed a similar pattern of expression of the *igf1*, demonstrated an exocrine/paracrine effect. Mohammed et al. (2015) described the gilt-head bream (*Sparus aurata*) muscle as one of the organs with highest expression *igf1r* followed by the pituitary gland. This is explained by the fact that fish, unlike mammals, possess a more active Igf-1 muscle binding, replacing the active insulin binding in mammalian muscles (Dai et al., 2015).

Peterson et al. (2005) demonstrated that GH injection did not have an effect on IGF expression in catfish (*Ictalurus punctatus*) muscle. Studies from Gabillard et al. (2003) and Reindl and Sheridan (2012) have shown that the GH-IGF system is modulated by adjusting peripheral sensitivity to both hormones as well as their availability at the target cells. In addition, nutritional state, insulin, somatostatin, cortisol and thyroid hormones, light and temperature exert effects on the expression and activity of the elements of the GH/IGF system. The interactions described above may explain the increase of *igf1a* and *igf1b* in the muscle even when the animals were kept at low temperatures since IGF mRNA expression is only partly regulated by GH in extra-hepatic tissues.

In general, changes in water temperature causes stress in fish, which includes physiological and behavioral adaptive changes and even death (Gordon, 2005). Our data for whole body cortisol showed remarkably higher amounts in animals kept at 23°C, with a noticeable reduction at early dark phase. The study conducted by Ramsay et al. (2009) in adult *D. rerio* demonstrated high levels of cortisol in animals stressed by handling, with a recovery of baseline levels 24 h post-stimulation. Unexpectedly, animals kept at 28°C had low hormonal levels at early morning and at night, not showing the well-known anticipatory peak in the early morning. Because of the time points chosen in our study, we suggest that the peak release of cortisol in animals kept at 28°C may have occurred prior to EL and that the high levels in animals at lower temperatures correspond to a sustained stress-induced response, with a phase shift of the peak.

Several studies using corticosteroids demonstrated a remarkable transcriptional modulation of clock genes (Zhao et al., 2016, 2018; Sousa et al., 2017). The fludrocortisone acetate at concentration of 6 and 42 μg/L downregulates the expression of *per1 per2* and *nr1d1* genes in peripheral tissues of adult zebrafish (*Danio rerio*) (Zhao et al., 2016). On the other hand, an increase in *per1* and *cry1b* transcripts was found in *D. rerio* ZEM-2S cells treated with 3 nM dexamethasone Sousa et al., 2017). In addition, in *D. rerio* eleuthero-embryos, progesterone, dexamethasone and 7α-ethinylestradiol at 1 μM up-regulate the expression of *per1, cry1a, rora, nr1d1*, and *nr1d2a* genes (Zhao et al., 2018). In contrast, our *in vivo* data suggest that the increase of the cortisol level (to 1.5 μg/g body weight) in animals at lower temperatures did not affect the expression of clock genes. We hypothesize that the interaction between lower temperature and the subsequent increase of cortisol may have resulted in a different response than when glucocorticoid is applied in a controlled temperature condition.

As to the expression of *gr*, there was a reduction in the mRNA of this receptor in the liver, but not in the muscle, of animals kept in colder temperature at early dark phase. Reports have shown that the activation of the hypothalamus-hypophysis-adrenal axis in heat stress-induced responses was accompanied by a peripheral adaptation in the amount and affinity of glucocorticoid receptors (Molijn et al., 1995). In our protocol (6 days at 23°C), it would be energy consuming to the organism to generate and keep high levels of *gr* mRNA and/or protein, as it would trigger an exacerbated response to cortisol which was elevated, and consequently could lead to homeostasis disruption.

In particular, temperature can have a strong impact on melatonin biosynthesis. The maximum activity of Aanat2 in a number of species correlated with the species-specific preferred water temperature and it is proposed as a mechanism of evolutionary adaptation (Falcón et al., 2009; Cowan et al., 2017). Furthermore, recent work in salmonids has suggested that pineal photoreceptors may also be thermoreceptors, since they express thermotransient receptor potential (TRP) channels (Nisembaum et al., 2015). In tropical fish such as the Senegalese sole (*Solea senegalensis*), the highest values of melatonin were observed at 25*^o^*C when the animals were kept in water ranging from 11.5*^o^*C to at 25°C compared to temperature ranging between 17 and 20°C (Vera et al., 2007). In our study, lower temperature led to a considerable increase on brain *aanat* levels in ED, suggesting possible increased melatonin levels. Studies demonstrated the rhythmic secretion of melatonin in *D. rerio* and its maximal release at the second third of the dark phase (Appelbaum et al., 2009; Ben-Moshe et al., 2016). Considering that at 28°C EL and ED, *aanat* expression exhibited no difference, we suggest that the highest *aanat* level and consequently the melatonin peak may have occurred at a nighttime point subsequent to the one evaluated. If this hypothesis holds true, there was an advance phase shift in the expression of *aanat2* in the brain at 23°C.

Loganathan et al. (2018) found that fish challenged by high temperatures had an increase in *mtnr1aa* and *mtnr1bb* mRNA levels in the brain. Interestingly, we found that the expression of *mtnr1aa* depends on the time point but not the temperature. As for *mtnr1bb*, we found a decrease of transcripts related to time point in the temperature control group, and an interaction between time and temperature shown by the ANOVA analysis. Likewise, our data suggest expression peaks of brain *mtnr1aa* in the dark, like the results of Yumnamcha et al. (2017) in *Danio rerio* females.

Melatonin is known to affect the rhythm of three clock genes *in vivo*, specifically *per1, per2* and *cry1* in the Japanese flounder (*Paralichthys olivaceus*) (Mogi et al., 2017). The activation of the melatonin receptor 1 inhibits cAMP formation, protein kinase A (PKA) activity, and phosphorylation of the cAMP-responsive element binding protein (CREB), required for the transcription of clock genes such as *per1* (Masana and Dubocovich, 2001).

*Danio rerio* cells exhibited decreased *per1* and *cry1-3* expression at high temperature and increased mRNA at low temperature (Lahiri et al., 2005). Curiously, previous studies in our laboratory have shown increased expression of *per1, per2, cry1a*, and *cry1b* clock genes after heat shock (33*^o^*C) in cells of *D. rerio* maintained in dark-light cycle; in this study the authors suggested that the transient receptor potential TRPV1 played a role in heat-mediated responses since *per2* increase was partially inhibited by TRPV1 blocker, demonstrating the channel participation in clock gene regulation by heat shock (Jerônimo et al., 2017).

Overall, we found higher expression of clock genes at EL with a marked decrease at ED and/or lower temperature. Several authors described a higher expression of the clock genes *per1, per2, cry1a*, and *cry1b* in *D. rerio* and other teleosts during the light phase or in the dark-light transition. In the specific case of *per2* and *cry1a*, the light stimulus is sufficient to trigger their expression, generally at ZT0 (night-day transition) (Delaunay et al., 2003; Wang, 2008). Our data corroborate the previous results: independently of the organ, the peak expression was at early light phase, assuming that *per* and *cry* transcription as well as the protein heterodimer formation (Per:Cry) were achieved; during early dark phase, smaller expressions were obtained, corresponding to the decrease of the transcripts in the dark phase. These findings demonstrate that the molecular clock seems to show phase shift or a reduction of transcript amplitude in cold-exposed fish. To confirm this, a 24 h long gene expression analysis every 3 or 4 h is needed. It should be noticed that lower temperature decreases the clock genes transcripts even in the EL in most studies. In ectothermic vertebrates, low temperature dampens circadian rhythms of melatonin secretion and clock gene expression (Rensing and Ruoff, 2002; Vallone et al., 2007). Also, Prokkola et al. (2018) reported that a genome-wide decrease in transcriptional rhythms is due to the combination of decreasing day length and cold temperature in fish. Furthermore, these and our results contrast with those obtained in *Danio rerio* larvae exposed to low temperatures (16 and 12°C). The previous study demonstrated up-regulation of genes like *per1, per2, cry1a*, and *cry1b* in animals kept at reduced temperatures, suggesting the involvement of circadian clocks in the cold adaptation (Long et al., 2012, 2013). These controversial reports indicate that cold responses evolve during ontogeny and may have been lost in adults. Further studies should be carried out to demonstrate this point.

Our *in vivo* data at 23°C are similar to those *in vitro* assays previously obtained by our group. In embryonic cells of *Danio rerio* (ZEM-2S) challenged by a cold pulse (25.5°C) in constant dark, there was a decrease in *per1* and *cry1b* mRNAs six hours after the end of the pulse (unpublished data). We can reconcile the above data with the presence/absence of light, since in the natural environment, the thermophase (high temperature) usually coincides with the photophase, and the cryophase (low temperatures) coincides with the scotophase (López-Olmeda and Sánchez-Vázquez, 2011).

As the gene transcripts may not reflect the dynamics of the protein expression and only two time points have been addressed, further studies are necessary to investigate the endpoint physiological responses at other time points.

## Conclusion

We demonstrated that lower temperature negatively affects the transcripts of several genes including clock genes and genes of the melatonin, GH and cortisol pathways in zebrafish. This downregulation possibly impairs the physiological parameters of the fish. We also showed that, in animals exposed to lower temperatures, there is a physiological compensation for high levels of hormones such as cortisol, with reduction of the respective receptors. In addition, the clock molecular machinery possibly correlates with the other analyzed systems including the GH axis and the melatonin pathway. In the Figure 9 we raised some hypotheses of effects of low temperatures on the endocrine axis and molecular system. For example, we hypothesize an increase in melatonin synthesis when animals are submitted at reduced temperatures modifying its receptors (Gi protein-coupled receptors). Thus, it may lead to an inhibition of adenylate cyclase activity which ultimately may results in the inhibition of CREB phosphorylation (transcription factor required for clock gene expression). We also suggest that if low temperature period was longer maybe a possible decrease of growth were observed, since the GH axis were inhibited. On the other hand, the reduction of clock transcripts can lead to a putative inhibition of genes as *gh1*. Consequently, this reduction in all organs analyzed can be associated to cold-modulation by melatonin and cortisol pathways or a direct effect of low temperature on clock gene transcripts. To prove these hypotheses more studies involving longer treatment and other experimental techniques are necessary.

## Data Availability Statement

The raw data supporting the conclusions of this article will be made available by the authors, without undue reservation.

## Ethics Statement

The animal study was reviewed and approved by Ethics Committee on Use of Animals (CEUA) of the Institute of Biosciences, University of São Paulo, protocol N° 331/2018.

## Author Contributions

CDSC, MNM, LVMA, and AMLC conceptualized the research. CDSC collected and analyzed the experimental data with the help of DDD, JASN, and OGL. CDSC wrote the manuscript, which was critically revised by AMLC, MNM, and LVMA. All the authors have approved the definitive version of the manuscript and agreed to be accountable for all aspects of the study in ensuring its accuracy and integrity.

## Conflict of Interest

The authors declare that the research was conducted in the absence of any commercial or financial relationships that could be construed as a potential conflict of interest.

## Publisher’s Note

All claims expressed in this article are solely those of the authors and do not necessarily represent those of their affiliated organizations, or those of the publisher, the editors and the reviewers. Any product that may be evaluated in this article, or claim that may be made by its manufacturer, is not guaranteed or endorsed by the publisher.

## Footnotes

1
http://www.ncbi.nlm.nih.gov/GenBank

## References

[B1] Alderman S. Bernier N. (2009). Ontogeny of the corticotropin-releasing factor system in zebrafish. *Gen. Comp. Endocrinol.* 164 61–69. 10.1016/j.ygcen.2009.04.007 19366623

[B2] Appelbaum L. Wang G. Maro G. Mori R. Tovin A. Marin W. (2009). Sleep-wake regulation and hypocretin-melatonin interaction in zebrafish. *Proc. Natl. Acad. Sci. U.S.A.* 106 21942–21947. 10.1073/pnas.906637106 19966231 PMC2799794

[B3] Aschoff J. (1965). Circadian rhythms in man. *Science* 148 1427–1432. 10.1126/science.148.3676.1427 14294139

[B4] Auró de Ocampo A. Ocampo L. (1999). Diagnóstico del estrés en peces. *Vet. Mex.* 30 337–344.

[B5] Azpeleta C. Martínez-Alvarez R. Delgado M. Isorna E. De Pedro N. (2010). Melatonin reduces locomotor activity and circulating cortisol in goldfish. *Horm. Behav.* 57 323–329. 10.1016/j.yhbeh.2010.01.001 20079741

[B6] Baltzegar D. Reading B. Douros J. Borski R. (2015). Role for leptin in promoting glucose mobilization during acute hyperosmotic stress in teleost fishes. *J. Endocrinol.* 220 61–72. 10.1530/JOE-13-0292 24194509

[B7] Ben-Moshe Z. Alon S. Vallone D. Bayleyen Y. Tovin A. Shainer I. (2016). Genetically blocking the zebrafish pineal clock affects circadian behavior. *PLoS Genet.* 12:e1006445. 10.1371/journal.pgen.1006445 27870848 PMC5147766

[B8] Boeuf G. Falcón J. (2001). Photoperiod and growth in fish. *Vie Milieu* 51 247–266.

[B9] Butler A. LeRoith D. (2001). Control of growth by the somatotrophic axis: growth hormone and the insulin-like growth factors have related and independent roles. *Annu. Rev. Physiol.* 63 141–164. 10.1146/annurev.physiol.63.1.141 11181952

[B10] Cowan M. Azpeleta C. López-Olmeda J. (2017). Rhythms in the endocrine system of fish: a review. *J. Comp. Physiol. B* 187 1057–1089. 10.1007/s00360-017-1094-5 28447151

[B11] Dai X. Zhang W. Zhuo Z. He J. Yin Z. (2015). Neuroendocrine regulation of somatic growth in fishes. *Sci. China Life. Sci.* 58 137–147. 10.1007/s11427-015-4805-8 25655896

[B12] Davies W. Tamai K. Zheng L. Fu J. Rihel J. Foster R. (2015). An extended family of novel vertebrate photopigments is widely expressed and displays a diversity of function. *Gen. Res.* 25 1666–1679. 10.1101/gr.189886.115 26450929 PMC4617963

[B13] Deane E. Woo N. (2009). Modulation of fish growth hormone levels by salinity, temperature, pollutants and aquaculture related stress: a review. *Rev. Fish. Biol. Fish.* 19 97–120. 10.1007/s11160-008-9091-0

[B14] Delaunay F. Thisse C. Thisse B. Laudet V. (2003). Differential regulation of period2 and period3 expression during development on the zebrafish circadian clock. *Gene Expr. Patterns* 3 319–324. 10.1016/S1567-133X(03)00050-4 12799078

[B15] Doyle S. Menaker M. (2007). Circadian photoreception in vertebrates. *Cold Spring Harb. Symp. Quant. Biol.* 72 499–508. 10.1101/sqb.2007.72.003 18419310

[B16] Egan R. Bergner C. Hart P. Cachat J. Canavello P. Elegante M. (2009). Understanding behavioral and physiological phenotypes of stress and anxiety in zebrafish. *Behav. Brain Res.* 205 38–44. 10.1016/j.bbr.2009.06.022 19540270 PMC2922906

[B17] Falcón J. Besseau L. Fuentès M. Sauzet S. Magnanou E. Boeuf G. (2009). Structural and functional evolution of the pineal melatonin system in vertebrates. *Ann. N. Y. Acad. Sci.* 1163 101–111. 10.1111/j.1749-6632.2009.04435.x 19456332

[B18] Falcón J. Besseau L. Sauzet S. Boeuf G. (2007). Melatonin effects on the hypothalamo-pituitary axis in fish. *Trends Endocrinol. Metab.* 18 81–88. 10.1016/j.tem.2007.01.002 17267239

[B19] Faught E. Aluru N. Vijayan M. (2016). The molecular stress response. *Fish Physiol.* 35 113–166. 10.1016/B978-0-12-802728-8.00004-7

[B20] Faught E. Vijayan M. (2016). Mechanisms of cortisol action in fish hepatocytes. *Comp. Biochem. Physiol. B Biochem. Mol. Biol.* 199 136–145. 10.1016/j.cbpb.2016.06.012 27445122

[B21] Gabillard J. Rescan P. Weil C. Fauconneau B. Le Bail P. (2003). Effects of temperature on GH/IGF system gene expression during embryonic development of rainbow trout (*Oncorhynchus mykiss*). *J. Exp. Zool.* 298 134–142. 10.1002/jez.a.10280 12884275

[B22] Gabillard J. Yao K. Vandeputte M. Guitierrez J. Le Bail P. (2006). Differential expression of two GH receptor mRNA following temperature change in rainbow trout (*Oncorhynchus mykiss*). *J. Endocrinol.* 190 29–37. 10.1677/joe.1.06695 16837608

[B23] Gesto M. Álvarez-Otero R. Conde-Sieira M. Otero-Rodi-o C. Usandizaga S. Soengas J. (2016). A simple melatonin treatment protocol attenuates the response to acute stress in the sole *Solea senegalensis*. *Aquaculture* 452 272–282. 10.1016/j.aquaculture.2015.11.006

[B24] Gordon J. (2005). *Temperature and Toxicology: And Integrative, Comparative, and Environmental Approach*, 1st Edn. Boca Raton, FL: CRC Press.

[B25] Grønkjær P. (2016). Otoliths as individual indicators: a reappraisal of the link between fish physiology and otolith characteristics. *Mar. Freshw. Res.* 67 881–888. 10.1071/MF15155

[B26] Hastings M. O’Neill J. Maywood E. (2007). Circadian clocks: regulators of endocrine and metabolic rhythms. *J. Endocrinol.* 195 187–198. 10.1677/JOE-07-0378 17951531

[B27] Jerônimo R. Moraes M. de Assis L. Ramos B. Rocha T. Castrucci A. (2017). Thermal stress in *Danio rerio*: a link between temperature, light, thermo-TRP channels, and clock genes. *J. Therm. Biol.* 68 128–138. 10.1016/j.jtherbio.2017.02.009 28689714

[B28] Kaneko M. Cahill G. (2005). Light-dependent development of circadian gene expression in transgenic zebrafish. *PLoS Biol.* 3:e34. 10.1371/journal.pbio.0030034 15685291 PMC546037

[B29] Klein S. Sheridan M. (2008). Somatostatin signaling and the regulation of growth and metabolism in fish. *Mol. Cell. Endocrinol.* 286 148–154. 10.1016/j.mce.2007.08.010 17919810

[B30] Lahiri K. Vallone D. Gondi S. Santoriello C. Dickmeis T. Foulkes N. (2005). Temperature regulates transcription in the zebrafish circadian clock. *PLoS Biol.* 3:e351. 10.1371/journal.pbio.0030351 16176122 PMC1233578

[B31] Leroith D. Bondy C. Yakar S. Liu J. Buter A. (2001). The somatomedin hypothesis: 2001. *Endocr. Rev.* 22 53–74. 10.1210/edrv.22.1.0419 11159816

[B32] Livak K. Schmittgen T. (2001). Analysis of relative gene expression data using real-time quantitative PCR and the 2(-delta delta C(T)) method. *Methods* 25 402–408. 10.1006/meth.2001.1262 11846609

[B33] Loeppky A. Belding L. Quijada-Rodriguez A. Morgan J. Pracheil B. Chakoumakos B. (2021). Influence of ontogenetic development, temperature, and pCO2 on otolith calcium carbonate polymorph composition in sturgeons. *Sci. Rep.* 11:13878. 10.1038/s41598-021-93197-6 34230512 PMC8260795

[B34] Loganathan K. Moriya S. Parhar I. (2018). High melatonin conditions by constant darkness and high temperature differently affect melatonin receptor mt1 and TREK channel trek2a in the brain of Zebrafish. *Zebrafish* 5, 473–483. 10.1089/zeb.2018.1594 30102584

[B35] Long Y. Li L. Li Q. He X. Cui Z. (2012). Transcriptomic characterization of temperature stress responses in larval Zebrafish. *PLoS One* 7:e37209. 10.1371/journal.pone.0037209 22666345 PMC3364249

[B36] Long Y. Song G. Yan J. He X. Li Q. Cui Z. (2013). Transcriptomic characterization of cold acclimation in larval Zebrafish. *BMC Genomics* 14:612. 10.1186/1471-2164-14-612 24024969 PMC3847098

[B37] López-Olmeda J. Sánchez-Vázquez F. (2011). Thermal biology of zebrafish (*Danio rerio*). *J. Therm. Biol.* 36 91–104. 10.1016/j.jtherbio.2010.12.005

[B38] Masana M. Dubocovich M. (2001). Melatonin receptor signaling: finding the path through the dark. *Sci. STKE* 107:pe39. 10.1126/stke.2001.107.pe39 11698691

[B39] Mogi M. Yokoi H. Suzuki T. (2017). Analyses of the cellular clock gene expression in peripheral tissue, caudal fin, in the Japanese flounder, *Paralichthys olivaceus*. *Gen. Comp. Endocrinol.* 248 97–105. 10.1016/j.ygcen.2017.02.009 28249777

[B40] Mohammed G. Martos J. Galal A. Mancera J. Martínez G. (2015). Insulin-like growth factor 1 (IGF-1) regulates prolactin, growth hormone, and IGF-1 receptor expression in the pituitary gland of the gilthead sea bream *Sparus aurata*. *Fish Physiol. Biochem.* 42 365–377. 10.1007/s10695-015-0144-8 26486515

[B41] Molijn G. Koper J. van Uffelen C. de Jong F. Brinkmann A. Bruinlng H. (1995). Temperature-induced down-regulation of the glucocorticoid receptor in peripheral blood mononuclear leucocyte in patients with sepsis or septic shock. *Clin. Endocrinol.* 43 197–203. 10.1111/j.1365-2265.1995.tb01915.x 7554315

[B42] Ngasainao M. Lukram I. (2016). A review on melatonin and its prospects in fish aquaculture. *J. Zool. Sci.* 4 34–41.

[B43] Nisembaum L. Besseau L. Paulin C. Charpantier A. Martin P. Magnanou E. (2015). In the heat of the night: thermo-TRPV channels in the salmonid pineal photoreceptors and modulation of melatonin secretion. *Endocrinology* 156 4629–4638. 10.1210/en.2015-1684 26389691

[B44] Oliveira J. Silveira M. Chacon D. Luchiari A. (2015). The zebrafish world of colors and shapes: preference and discrimination. *Zebrafish* 12 166–173. 10.1089/zeb.2014.1019 25674976

[B45] Peterson B. Waldbieser G. Bilodeau L. (2005). Effects of recombinant bovine somatotropin on growth and abundance of mRNA for IGF-I and IGF-II in channel catfish (*Ictalurus punctatus*). *J. Anim. Sci.* 83 816–824. 10.2527/2005.834816x 15753336

[B46] Prokkola J. Nikinmaa M. Lewis M. Anttila K. Kanerva M. Ikkala K. (2018). Cold temperature represses daily rhythms in the liver transcriptome of a stenothermal teleost under decreasing day length. *J. Exp. Biol.* 221:jeb170670. 10.1242/jeb.170670 29361589

[B47] Quera S. Hartley S. (2012). Mood disorders, circadian rhythms, melatonin and melatonin agonists. *J. Cent. Nerv. Syst. Dis.* 4 15–26. 10.4137/JCNSD.S4103 23650464 PMC3619438

[B48] Ramsay J. Feist G. Varga Z. Westerfield M. Kent M. Schreck C. (2009). Whole-body cortisol response of zebrafish to acute net handling stress. *Aquaculture* 297 157–162. 10.1016/j.aquaculture.2009.08.035 25587201 PMC4289633

[B49] Reindl K. M. Sheridan M. A. (2012). Peripheral regulation of the growth hormone-insulin-like growth factor system in fish and other vertebrates. *Comp. Biochem. Physiol. A Mol. Integr. Physiol.* 163 231–245. 10.1016/j.cbpa.2012.08.003 22909791

[B50] Reindl K. Kittilson J. Bergan H. Sheridan M. (2011). Growth hormone-stimulated insulin-like growth factor-1 expression in rainbow trout (*Oncorhynchus mykiss*) hepatocytes is mediated by ERK, PI3K-AKT, and JAK-STAT. *Am. J. Physiol. Regul. Integr. Comp. Physiol.* 301 236–243. 10.1152/ajpregu.00414.2010 21490369

[B51] Rensing L. Ruoff P. (2002). Temperature effect on entrainment, phase shifting, and amplitude of circadian clocks and its molecular bases. *Chronobiol. Int.* 19 807–864. 10.1081/CBI-120014569 12405549

[B52] Rudy D. Forsberg J. (2016). “Chapter 5: Biology and ecosystem research. Chapter 5.2: An examination of otolith growth increments in relation to somatic growth for Pacific halibut (Hippoglossus stenolepis),” in *Report Assessment and Research Activities*. (Seattle, WA: International Pacific Halibut Commission).

[B53] Sánchez-Vázquez F. López-Olmeda J. Vera L. Migaud H. López-Patiño M. Míguez J. (2019). Environmental cycles, melatonin, and circadian control of stress response in fish. *Front. Endocrinol.* 10:279. 10.3389/fendo.2019.00279 31244768 PMC6579845

[B54] Sousa J. Magalhaes K. Da Silveira Cruz Machado S. Moraes M. Castrucci A. (2017). Dexamethasone modulates non-visual opsins, glucocorticoid receptor and clock genes in *Danio rerio* ZEM-2S cells. *Biomed Res. Int.* 2017:8459385. 10.1155/2017/8459385 28589149 PMC5446867

[B55] Torres-Farfan C. Richter H. Rojas P. Vergara M. Forcelledo M. Valladares L. (2003). mt1 Melatonin receptor in the primate adrenal gland: inhibition of adrenocorticotropin-stimulated cortisol production by melatonin. *J. Clin. Endocrinol. Metab.* 88 450–458. 10.1210/jc.2002-021048 12519889

[B56] Vallone D. Frigato E. Vernesi C. Foá A. Foulkes N. Bertolucci C. (2007). Hypothermia modulates circadian clock gene expression in lizard peripheral tissues. *Am. J. Physiol. Regul. Integr. Comp. Pysiol.* 292 R160–R166. 10.1152/ajpregu.00370.2006 16809482

[B57] Vallone D. Lahiri K. Dickmeis T. Foulkes N. (2005). Zebrafish cell clocks feel the heat and see the light! *Zebrafish* 2 171–187. 10.1089/zeb.2005.2.171 18248192

[B58] Vatine G. Vallone D. Gothilf Y. Foulkes N. (2011). It’s time to swim! Zebrafish and the circadian clock. *FEBS Lett.* 585 1485–1494. 10.1016/j.febslet.2011.04.007 21486566

[B59] Vera L. De Oliveira C. López-Olmeda J. Ramos J. Mananos E. Madrid J. (2007). Seasonal and daily plasma melatonin rhythms and reproduction in *Senegal sole* kept under natural photoperiod and natural or controlled water temperature. *J. Pineal Res.* 43 50–55. 10.1111/j.1600-079X.2007.00442.x 17614835

[B60] Wang H. (2008). Comparative analysis of period genes in teleost fish genomes. *J. Mol. Evol.* 67 29–40. 10.1007/s00239-008-9121-5 18535754

[B61] Wang J. Song Q. Yu D. Yang G. Xia L. Su K. (2015). Ontogenetic development of the auditory sensory organ in zebrafish (*Danio rerio*): changes in hearing sensitivity and related morphology. *Sci. Rep.* 5:15943. 10.1038/srep15943 26526229 PMC4630651

[B62] West A. Bechtold D. (2015). The cost of circadian desynchrony: evidence, insights and open questions. *Bioessays* 37 777–788. 10.1002/bies.201400173 26010005 PMC4973832

[B63] White C. Alton L. Frappell P. (2012). Metabolic cold adaptation in fishes occurs at the level of whole animal, mitochondria and enzyme. *Proc. Biol. Sci.* 279 1740–1747. 10.1098/rspb.2011.2060 22158960 PMC3297453

[B64] Yamamoto T. Nakahata Y. Tanaka M. Yoshida M. Soma H. Shinohara K. (2005). Acute physical stress elevates mouse period1 mRNA expression in mouse peripheral tissues via a glucocorticoid-responsive element. *J. Biol. Chem.* 280 42036–42043. 10.1074/jbc.M509600200 16249183

[B65] Yumnamcha T. Ahmad Z. Rajiv C. Dharmajyoti D. Mondal G. Sanjita H. (2017). Interaction of melatonin and gonadotropin-inhibitory hormone on the Zebrafish brain-pituitary axis. *Mol. Reprod. Dev.* 84 389–400. 10.1002/mrd.22795 28295807

[B66] Zhang E. Liu Y. Dentin R. Pongsawakul P. Liu A. Hirota T. (2010). Cryptochrome mediates circadian regulation of cAMP signaling and hepatic gluconeogenesis. *Nat. Med.* 16 1152–1156. 10.1038/nm.2214 20852621 PMC2952072

[B67] Zhao Y. Zhang K. Fent K. (2016). Corticosteroid fludrocortisone acetate targets multiple end points in zebrafish (*Danio rerio*) at low concentrations. *Environ. Sci. Technol.* 50 10245–10254. 10.1021/acs.est.6b03436 27618422

[B68] Zhao Y. Zhang K. Fent K. (2018). Regulation of zebrafish (*Danio rerio*) locomotor behavior and circadian rhythm network by environmental steroid hormones. *Environ. Pollut.* 232 422–429. 10.1016/j.envpol.2017.09.057 28993021

