# Supplementary material for: Low Temperature Effect on the Endocrine and Circadian Systems of Adult Danio rerio
Source: Front Physiol. 2021 Nov 24;12:707067. doi: 10.3389/fphys.2021.707067 (PMC8652057; doi:10.3389/fphys.2021.707067)
Supplement: Supplementary file 1 [file Data_Sheet_1.docx]

**SUPPLEMENTARY MATERIAL**

**Table S1. Distribution of fish for the experiments**

| **qPCR group: a total of 96 animals** | | | | | |
| --- | --- | --- | --- | --- | --- |
| Tissue | Temperature | Total number of animals | Number of tissue samples per pool | Total number of pools | Pools used |
| Brain | 28^o^C | 48 at 28°C  48 at 23°C | 4 | 12 | n=3-6 at EL n=3-6 at ED |
|  | 23^o^C |  |  | 12 | n=3-6 at EL n=3-6 at ED |
| Liver | 28^o^C |  | 4 | 12 | n=3-6 at EL n=3-6 at ED |
|  | 23^o^C |  |  | 12 | n=3-6 at EL n=3-6 at ED |
| Muscle | 28^o^C |  | 4 | 12 | n=3-6 at EL n=3-6 at ED |
|  | 23^o^C |  |  | 12 | n=3-6 at EL n=3-6 at ED |
| **Cortisol group: a total of 96 animals** | | | | | |
| Tissue | Temperature | Total number of animals | Number of animals per pool | Total number of pools | Pools used |
| Whole body | 28^o^C | 48 | 4 | 12 | n=3-6 at EL n=3-6 at ED |
|  | 23^o^C | 48 |  | 12 | n=3-6 at EL n=3-6 at ED |

**Table S2. Weights (g) of the animals used for cortisol extraction**

| **28°C group** | | | | | | |
| --- | --- | --- | --- | --- | --- | --- |
|  | **ZT2** | | | **ZT16** | | |
| **Sample** | **1** | **2** | **3** | **4** | **5** | **6** |
| **Total weight** | 0.600 | 0.640 | 0.596 | 0.554 | 0.482 | 0.554 |
| **23°C group** | | | | | | |
|  | **ZT2** | | | **ZT16** | | |
| **Sample** | **1** | **2** | **3** | **4** | **5** | **6** |
| **Total weight** | 0.686 | 0.432 | 0.594 | 0.658 | 0.724 | 0.478 |

* Each sample corresponds to a pool of 4 animals
